# Parallel modulation of intracortical excitability of somatosensory and visual cortex by the gonadal hormones estradiol and progesterone

**DOI:** 10.1038/s41598-020-79389-6

**Published:** 2020-12-17

**Authors:** Nasim Schloemer, Melanie Lenz, Martin Tegenthoff, Hubert R. Dinse, Oliver Höffken

**Affiliations:** 1 Department of Neurology, Berufsgenossenschaftliches Universitätsklinikum Bergmannsheil GmbH, Ruhr-University Bochum, 44789 Bochum, Germany; 2 Department of Psychiatry, Medical Faculty, University of Cologne, 50931 Cologne, Germany; 3 Institute for Neuroinformatik, Neural Plasticity Lab, Ruhr-University of Bochum, 44780 Bochum, Germany

**Keywords:** Neuroscience, Neuronal physiology, Sensory processing, Sexual behaviour, Somatosensory system, Visual system

## Abstract

The levels of the gonadal hormones estradiol and progesterone vary throughout the menstrual cycle thereby affecting cognition, emotion, mood, and social behaviour. However, how these hormones modulate the balance of neural excitation and inhibition, which crucially regulate processing and plasticity, is not fully understood. We here used paired-pulse stimulation to investigate in healthy humans the action of low and high estradiol and progesterone on intracortical inhibition in somatosensory (SI) and visual cortex (V1). We found that paired-pulse suppression in both SI and VI depended on estradiol. During high estradiol levels, paired-pulse suppression was significantly reduced. No comparable effects were found for progesterone, presumably due to a confounding effect of estradiol. Also, no hormone level-depending effects were observed for single-pulse evoked SEPs (somatosensory evoked potentials) and VEPs (visual evoked potentials) indicating a specific hormonal action on intracortical processing. The results demonstrate that estradiol globally modulates the balance of excitation and inhibition of SI and VI cortex.

## Introduction

There is agreement that alterations of synaptic transmission are based on a delicate balance between excitatory and inhibitory processes^1,2^. A central role is played by the inhibitory neurotransmitter γ-aminobutyric acid (GABA), which is involved in numerous mechanisms that act to stabilize and to control neural excitability. GABAergic circuits are also critically involved during learning, where homeostatic plasticity mechanisms ensure this precise balance between excitation and inhibition^3–6^.

Estrogen and progesterone are known to exhibit opposite roles on excitatory and inhibitory receptors. While estrogen augments *N*-methyl-d-aspartate- (NMDA)-mediated glutamate receptor activity by suppressive effects on GABA release, progesterone enhances GABA-mediated neurotransmission through its action at GABA_A_ receptors, and inhibits neural excitation by suppression of excitatory glutamate response. A major indirect action of progesterone is induced after being metabolized to neuroactive steroids^7–11^.

Given the modulating role of gonadal hormones on excitation and inhibition, strong implications are to be expected on brain functions. In fact, numerous studies have shown that physiological functions, cognition, emotion, mood, sexual, and social behaviour is modulated through the menstrual cycle^12–18^. However, less is known about the impact of gonadal hormones on intracortical excitability, which are critically parameters involved in controlling processing, plasticity and learning^19,20^.

To measure excitation and inhibition in human subjects, the assessment of paired-pulse behaviour has become a standard procedure. It consists of application of pairs of stimuli in close succession (paired-pulse stimulation), which can be used as a marker of intracortical excitability in sensory cortices. This approach is somewhat equivalent to paired-pulse transcranial magnetic stimulation (TMS), which is widely used to assess plastic changes in human motor cortex (cf.^21,22^ for review).

**Figure 1:**
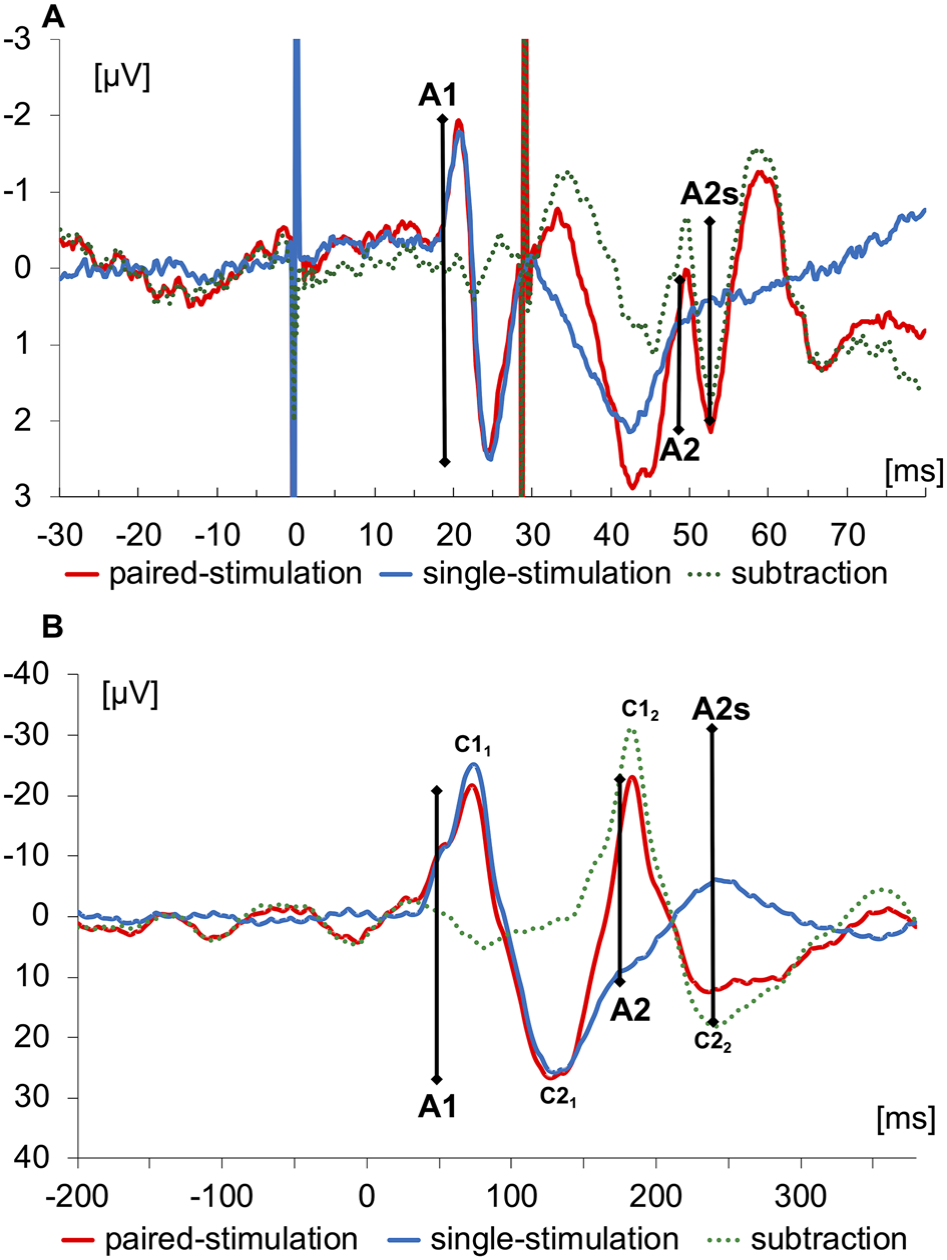
Analysis of paired-stimulation evoked potentials. (**A**) Somatosensory evoked potentials (SEP) recorded over CP3 after single (blue trace) and paired-stimulation with an ISI of 30 ms (red trace) of right median nerve. The dotted green trace results from subtracting the single-stimulation trace from the paired-stimulation trace. The analysed amplitudes of the first response (A1) and second response (A2) of the N20-P25 component after paired-stimulation are marked by vertical bars; amplitudes of the second response after subtracting the response to a single stimulation are denoted as A2s. Artifacts resulting from electrical stimulation at 0 and 30 ms were truncated. (**B**) Visually evoked potentials (VEPs) recorded over cortical Oz of one subject after single (blue trace) and paired-stimulation with stimulation onset asynchrony (SOA) of 93 ms (red trace). The label C was used to characterize the positive and negative components of the first and second cortical response. The dotted green trace results from subtracting the single-stimulation trace from the paired-stimulation trace. The analysed amplitudes of the first response (A1 = C2_1_–C1_1_) and second response (A2 = C2_2_–C1_2_) amplitudes after paired stimulation are marked by vertical bars; amplitudes of the second response after subtracting the response to a single stimulation are labelled as amplitudes of the second (A2s).

When applied in sensory areas, paired-pulse suppression describes the outcome of stimulation with very short interstimulus intervals (ISI) that the cortical responses to the second stimulus are significantly suppressed compared to the first stimulus. Paired-pulse suppression is quantified by calculating the ratio of the amplitude of the second response divided by the amplitude of the first response. Small amplitude ratios indicate strong paired-pulse suppression, while large ratios indicate little paired-pulse suppression which is taken as a marker for enhanced excitation (Fig. 1).

Recordings made at different hierarchical levels of the sensory pathways have demonstrated that paired-pulse suppression is exclusively cortical, with no indication of suppression at thalamic or brain stem levels^23–25^. Pharmacological studies provided strong evidence that, comparable to motor cortex, suppression is GABA-mediated^26^. Because of these properties, and because paired-pulse stimulation can be easily applied, changes in paired-pulse suppression are widely used as indices of plastic or disease-related changes in sensory cortex. For example, reduced paired-pulse suppression has been found to be linked to a gain of behavioural and perceptual performance^27–31^. Furthermore, paired-stimulation suppression in somatosensory system has been studied to assess facilitatory and inhibitory effects in several neurological disorders (cf.^32–34^).

Several studies have addressed possible modulating effects throughout the menstrual cycle on human motor cortical excitability measures such as resting motor threshold, cortical silent period, intracortical inhibition and intracortical facilitation providing mixed results. Using transcranial magnetic stimulation (TMS) over primary motor cortex, it had been reported that the excitability in the motor cortex changes during the menstrual cycle^35^. Intracortical inhibition was reported to be more pronounced during the mid-luteal phase as compared to the follicular phase. Based on that, the authors assumed an inhibitory progesterone effect, although an antagonistic effect of estradiol could not be excluded. In a follow-up study, excitatory effects were found during the follicular phase suggesting an excitatory role of estradiol^36^. While an estradiol-associated excitatory effect was also reported for motor cortex excitability^37^, more recent studies failed to observe any changes of resting motor threshold, cortical silent period, intracortical inhibition and intracortical facilitation throughout the menstrual cycle^17,38^. More recently, a study demonstrated unchanged corticospinal excitability, but enhanced short-interval intracortical inhibition on day 21 compared with days 14 and 2^39^. Therefore, whether and how the menstrual cycle affects measures of cortical excitability remains controversial.

Despite these contradicting findings, a potential modulating role of female gonadal hormones on measures of motor cortex excitability appears highly conceivable. Even more, assuming a modulating role of intracortical inhibition through estradiol and progesterone, it appears plausible to expect parallel changes of excitability throughout the central nervous system. Most importantly, data about a possible hormonal modulation of intracortical excitability of sensory cortical areas are lacking, although an assessment of paired-pulse behaviour has been successfully applied in somatosensory^31^, visual^40^ and auditory cortex^41^.

Therefore, to demonstrate a non-local and parallel modulation of global cortical excitability by female gonadal hormones, we took advantage of the fact that paired-pulse suppression can be assessed in both the somatosensory and visual cortex. To this aim, based on measurements on four time points during the menstrual cycle, we assessed in healthy women without any use of hormonal contraception their minimal and maximal levels of estradiol and progesterone. In parallel, we assessed paired-pulse suppression in primary somatosensory and visual cortex. As a control, paired-pulse suppression data were compared to measurements in age-matched men. We found that paired-pulse suppression in both somatosensory and visual cortex depended on estradiol, but not on progesterone.

## Results

**Figure 2:**
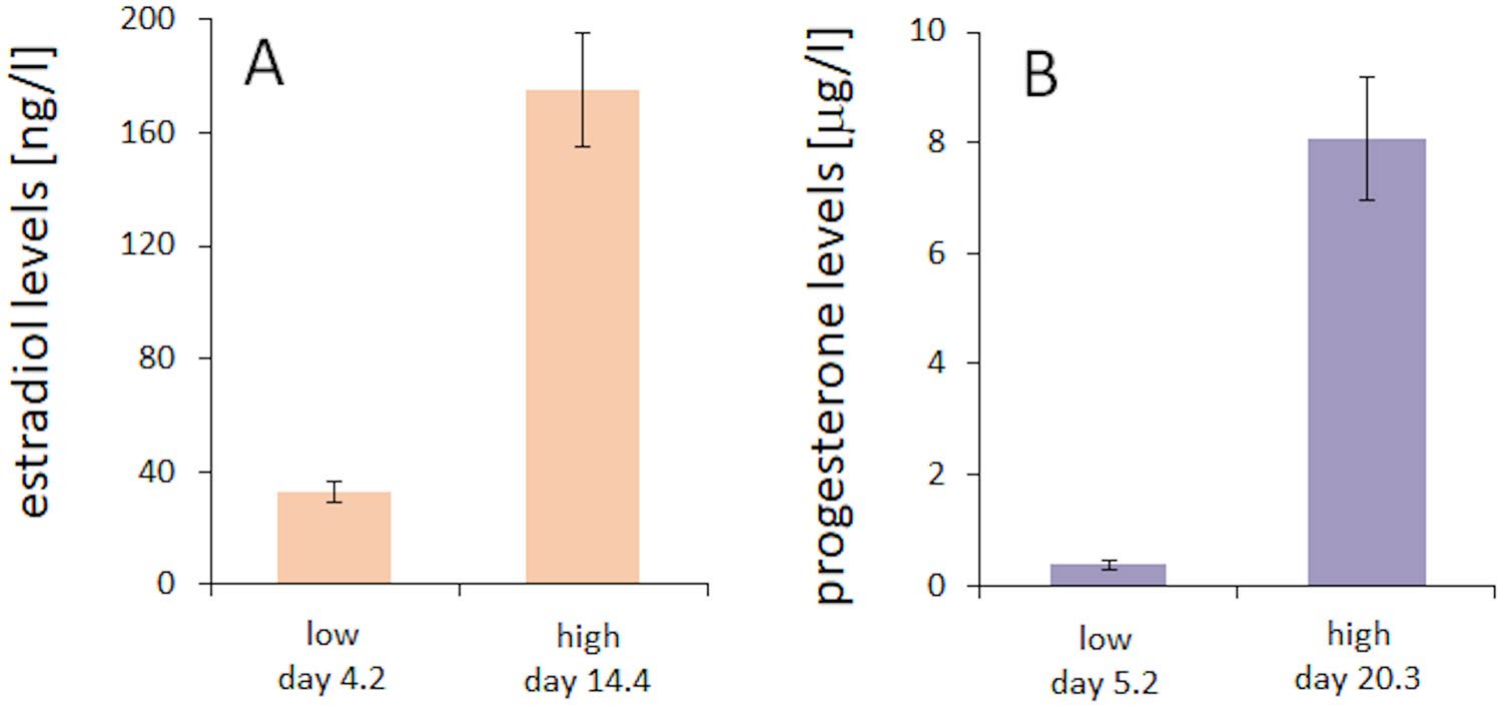
Minimal and maximal hormone levels. Average levels of minimal and maximal estradiol (**A**) and progesterone (**B**) levels. Data are represented as mean ± SEM.

**Figure 3:**
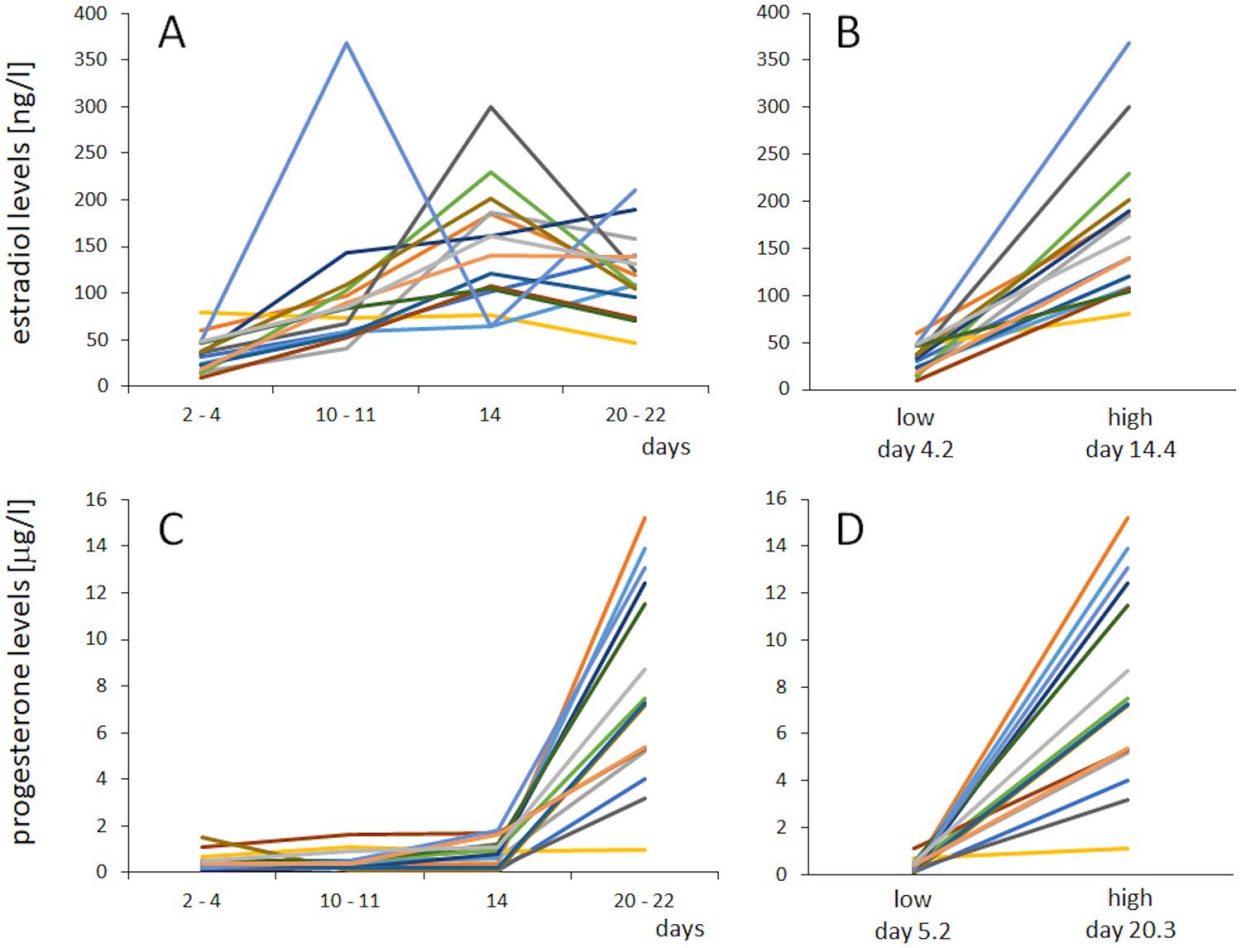
Individual hormone levels. Hormone levels of estradiol (**A**) and progesterone (**C**) at the testing time points (day 2–4, day 10–11, day 14, and day 20–22), and at the individual lowest and highest levels of estradiol (**B**) and progesterone (**D**).

As a first step, we analysed in the group of women the levels of the gonadal steroid hormones estradiol and progesterone. Based on these data, we then calculated the minimal and the maximal estradiol and progesterone levels for each participant independent of the time point within the menstrual cycle. Average estradiol levels were 32.7 ng/l (SD 14.9) at the time of minimum estradiol, and 174.9 ng/l (SD 78.4) at the time of maximum estradiol (Fig. 2A). Average progesterone levels at the time of minimum progesterone were 0.37 µg/l (SD 0.27), and 8.07 µg/l (SD 4.26) at the time of maximal progesterone (Fig. 2B). All single subject hormone data are shown in Fig. 3.

### Effects of minimal and maximal hormone levels on paired-pulse suppression

**Figure 4:**
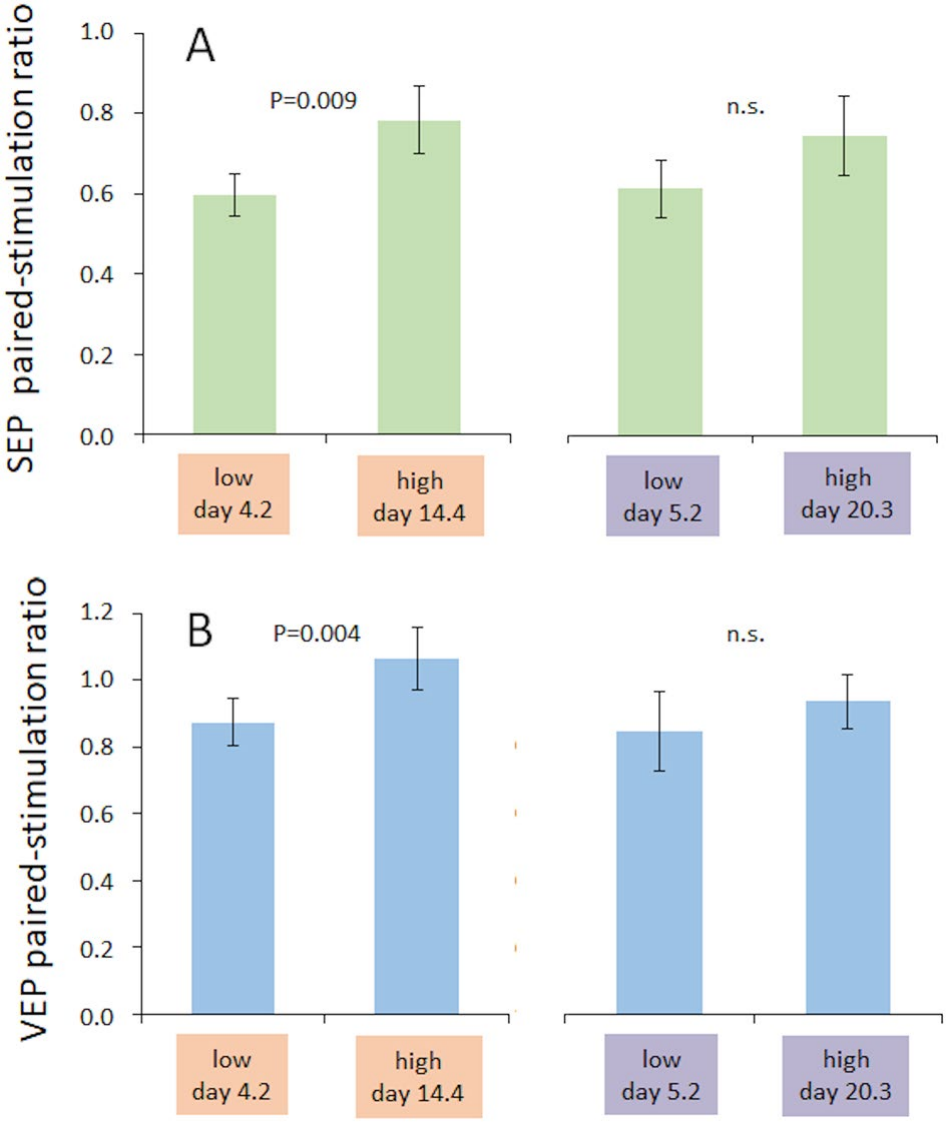
Effects of gonadal hormones on intracortical inhibition. Paired-stimulation ratios of SEP (**A**) and VEP (**B**) at individual lowest and highest levels of estradiol (left) and progesterone (right). Data are represented as mean ± SEM.

Based on the above analysis, we then compared the paired-pulse suppression data from paired SEP- and VEP-recordings (somatosensory evoked potentials, visual evoked potentials) performed at the time of minimum and maximum estradiol and progesterone levels each. (Fig. 4). Paired-stimulation ratios for paired-SEP recordings differed significantly between time points at minimal (mean 0.59, SEM 0.05) and maximal estradiol levels (mean 0.78, SEM 0.08; rmANOVA F(1,14) = 9.057, p = 0.009, partial η^2^ = 0.393). In contrast, for minimal (mean paired-stimulation ratio 0.61, SEM 0.07) and maximal progesterone levels (mean paired-stimulation ratio 0.74, SEM 0. 01), no differences in paired-stimulation SEP ratios were found (rmANOVA F(1,14) = 1,481, p = 0.244, partial η^2^ = 0.096), see Fig. 4A.

**Figure 5:**
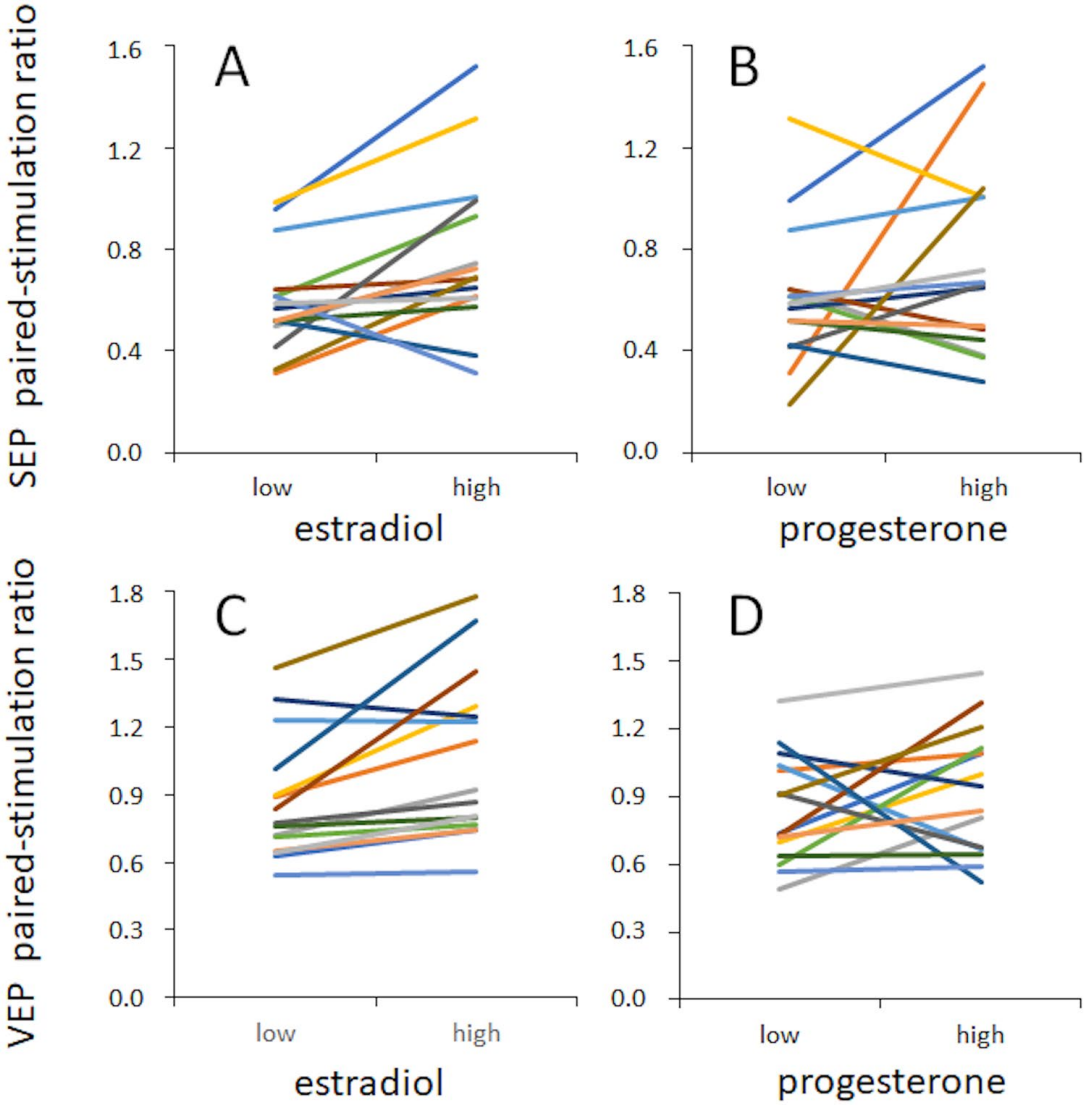
Individual paired-stimulation ratios. Paired-stimulation ratios of SEPs and VEPs recorded at the individual lowest and highest levels of estradiol (**A**,**B**) and progesterone (**C**,**D**).

**Figure 6:**
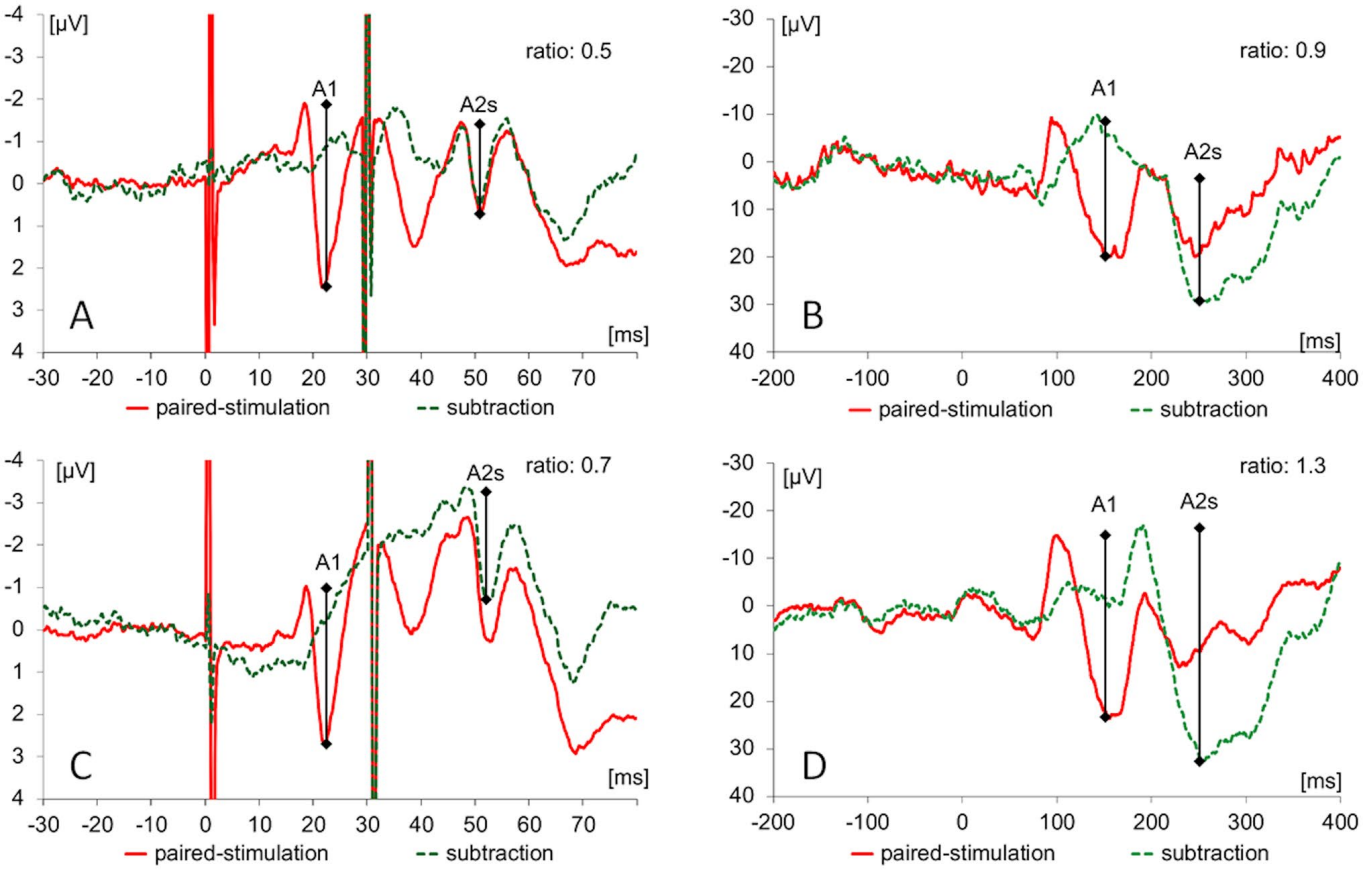
Paired SEP and VEP traces from one subjects. Shown are SEPs (**A**,**C**) and VEPs (**B**,**D**) recorded at lowest (**A**,**B**) and highest levels (**C**,**D**) of estradiol. Original traces obtained during paired stimulation are shown in red, while the dotted green traces result from subtracting the single-stimulation trace from the paired-stimulation trace (cf. Figure 1). The response amplitudes of the first response (A1) and the second response after subtraction (A2s) and the resulting paired-stimulation ratios are shown.

Paired pattern onset/offset VEP recordings showed a similar result. Paired-stimulation ratios differed significantly between time points at minimal (mean 0.87, SEM 0.07) and maximal estradiol levels (mean 1.06, SEM 0.098; rmANOVA F(1,14) = 11.632, p = 0.004, partial η^2^ = 0.454). For minimal (mean paired pulse ratio 0.93, SEM 0.118) and maximal progesterone levels (mean paired-stimulation ratio 0.97, SEM 0.082), no differences in paired-stimulation SEP ratios were found (rmANOVA F(1,14) = 1.088, p = 0.315, partial η^2^ = 0.072), see Fig. 4B. Calculation of the effect sizes (Cohens d) based on the partial η^2^ in our data indicates that the effects of hormones on excitability are large (0.80 for paired SEPs, and 0.919 for paired VEPs). All single subjects data are shown in Fig. 5. In addition, representative traces of SEPs and VEPs recorded from a subject during low and high estradiol levels are shown in Fig. 6.

### Relation between estradiol and progesterone levels

**Figure 7:**
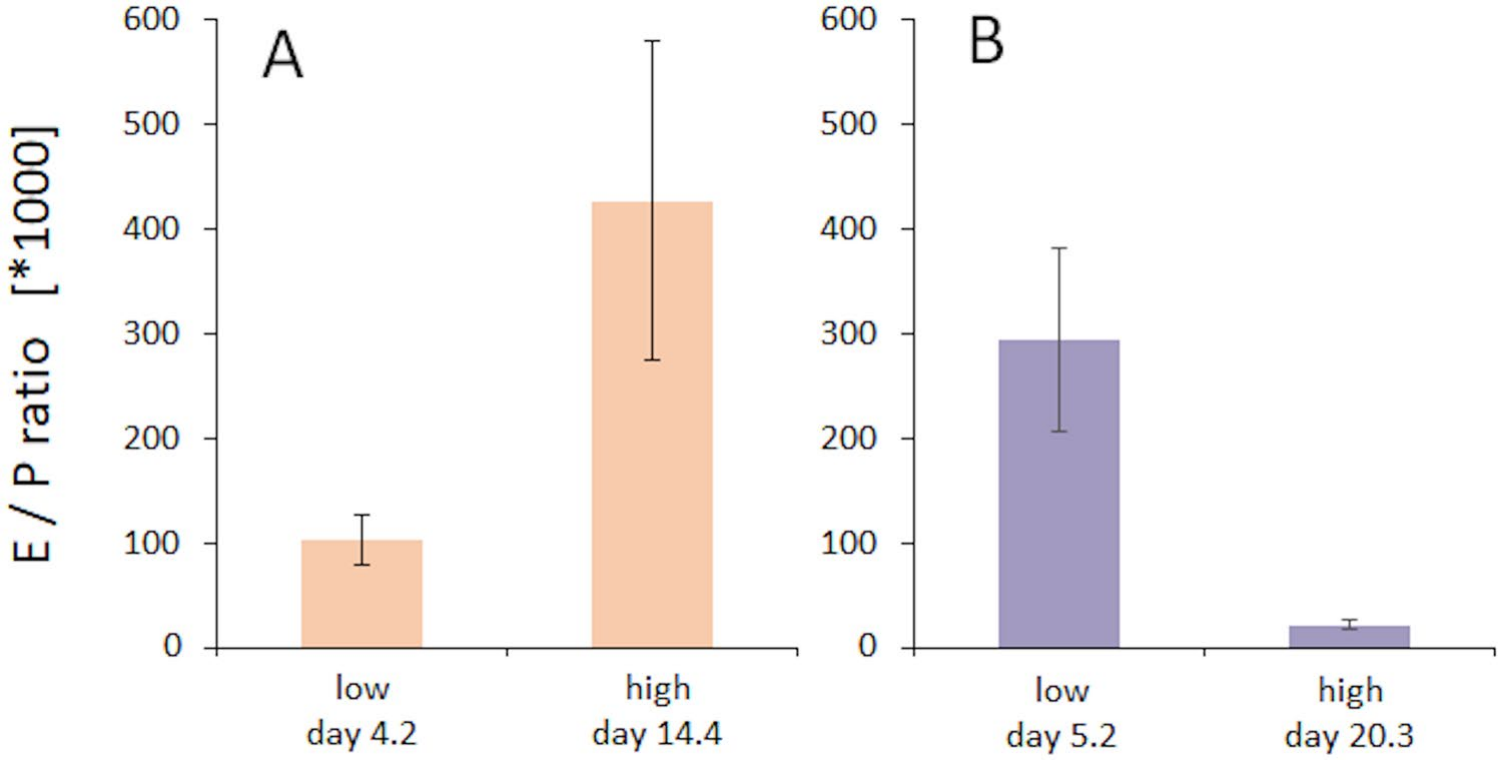
Estradiol to progesterone ratios. Average ratios between estradiol and progesterone (E/P ratio) at times of minimal and maximal estradiol (**A**) and minimal and maximal progesterone (**B**) levels. Data are represented as mean ± SEM.

**Figure 8:**
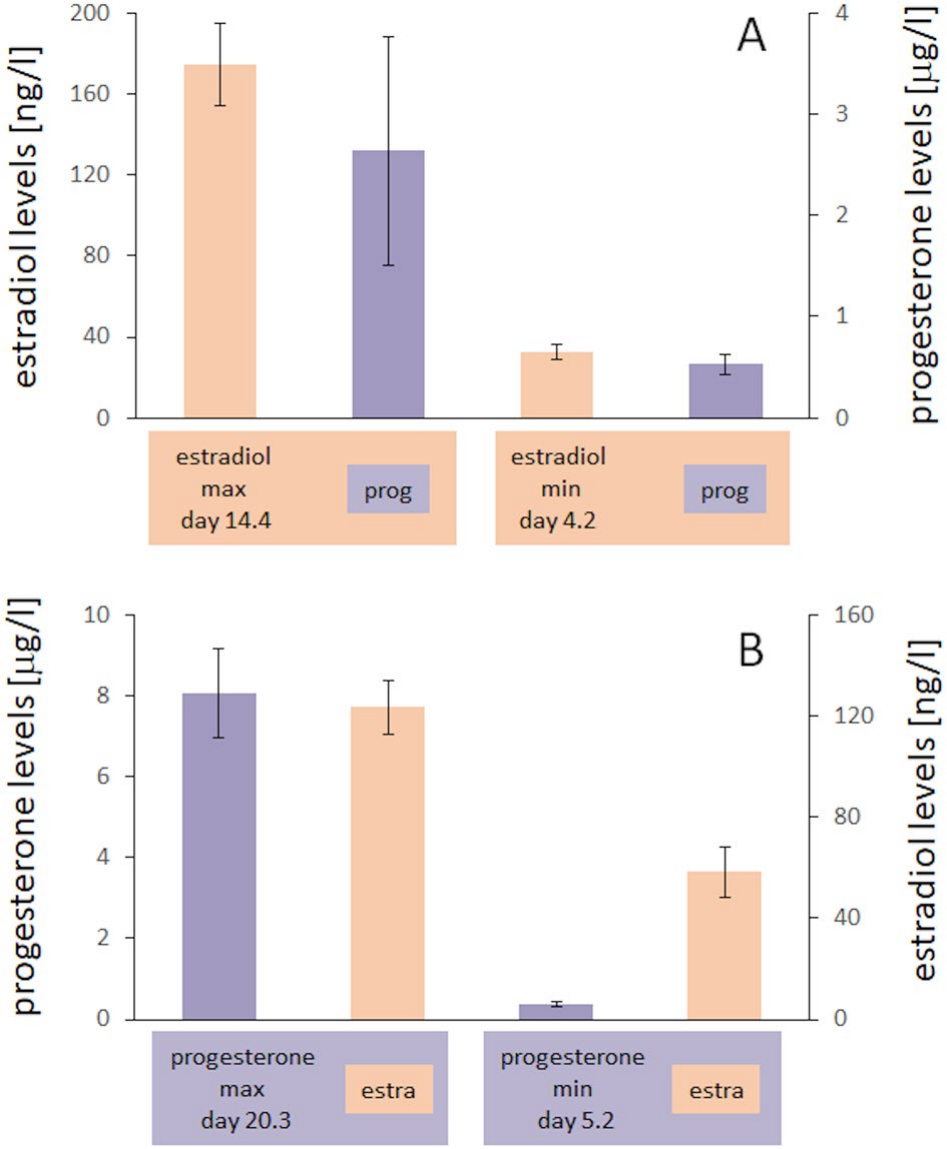
Relation between estradiol and progesterone levels. Hormone levels for estradiol and progesterone at lowest and highest levels for estradiol (**A**) and for progesterone (**B**). *estra* estradiol, *prog* progesterone. Data are represented as mean ± SEM.

Our data thus show that elevated estradiol levels are associated with enhanced cortical excitability as indicated by reduced paired-pulse suppression, but no comparable effects were seen for progesterone. However, during the luteal phase, both estradiol and progesterone are elevated. To further analyse the relation between both hormones and their possible interaction, we first calculated the estradiol-progesterone ratios as shown in Fig. 7A,B, and correlated the minimal and maximal ratios with the paired-pulse suppression obtained for SI and VI recordings. We found no significant correlation and only marginal variance explained (paired SEP: p = 0.854, r = − 0.024; paired VEPs: p = 0.066, r = 0.243). This finding indicates that estradiol-progesterone ratios provide only limited insight into paired-pulse suppression obtained in sensory cortical areas. As a next step we therefore analysed possible confounds arising from potential joint effects of both hormones. For estradiol, we found that for the time points of both maximal and minimal estradiol levels the parallel levels of progesterone were low (Fig. 8A), with no differences in their means (rmANOVA F(1,14) = 3.271, p = 0.092, partial η^2^ = 0.189). On the other hand, for progesterone, during the time points of maximal and minimal progesterone, the estradiol levels differed significantly as well (Fig. 8B), with high estradiol levels paralleling high progesterone, and vice versa (mANOVA F(1,14) = 18.106, p = 0.01, partial η^2^ = 0.564). However, despite the significant differences of both hormone levels, no differences for paired-pulse suppression were observed. These data suggest that the high estradiol levels simultaneously present during high progesterone might have masked any suppressive effects of progesterone.

### Comparing paired-pulse suppression in men and women

To obtain insight in how far paired-pulse suppression differs at different time points between men and women, we separately compared men and women during maximal and minimal estradiol levels.

For paired SEPs recordings, we found no differences between men (mean 0.79, SEM 0.07) and female (mean 0.78, SEM 0.08) during maximal estradiol (F(1,28) = 0.000, p = 0.983, partial η^2^ = 0.000). However, there were significant differences between men and female (mean 0.59, SEM 0.05) during minimal estradiol (F(1,28) = 4.945, p = 0.034, partial η^2^ = 0.150). This finding implies that the values found in men and in women during high estradiol levels, are comparable. For paired VEPs recordings, however, we found no differences between men (mean 0.95, SEM 0.065) and female (mean 1.06, SEM 0.098) during maximal estradiol levels (F(1,28) = 1.144, p = 0.294, partial η^2^ = 0.039), and no differences between men and female (mean 0.87, SEM 0.07) during minimal estradiol levels (F(1,28) = 0.543, p = 0.467, partial η^2^ = 0.019).

### Hormone effects on single stimulation evoked potentials

**Figure 9:**
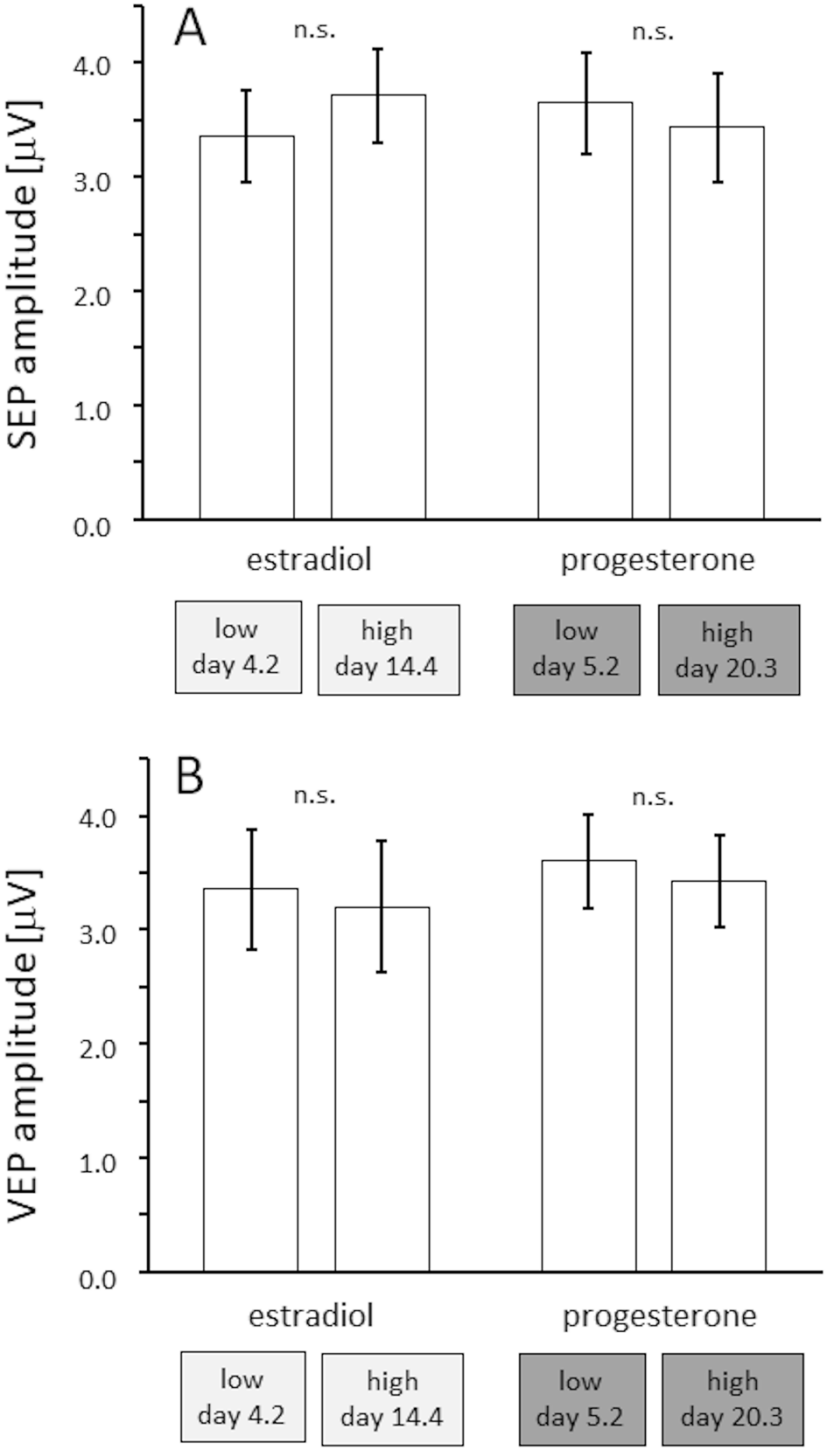
Effects of gonadal hormones on evoked potentials. Effect of lowest and highest levels of estradiol and progesterone on single-stimulation SEP (**A**) and VEP (**B**). Data are represented as mean ± SEM.

We also analysed whether the single-stimulation EPs were affected by low and high levels of gonadal steroid hormones. No effects were observed for single-stimulation SEPs (estradiol: F(1,14) = 1.142, p = 0.303, partial η^2^ = 0.075; progesterone: F(1,14) = 0.582, p = 0.458, partial η^2^ = 0.040) (Fig. 9A) and for single-stimulation VEPs (estradiol: F(1,14) = 0.228, p = 0.641, partial η^2^ = 0.016; progesterone: F(1,14) = 0.213, p = 0.651, partial η^2^ = 0.015; Fig. 9B). These results indicate that the amplitudes of sensory stimulation evoked potentials in both the somatosensory and the visual system remain unaffected by gonadal steroid hormones, but that the nature of intracortical processing as marked by paired pulse suppression was shifted towards higher excitability.

### Relation between paired-pulse suppression and hormone levels

Pearson’s correlation calculation indicated a lack of significant correlations between paired-stimulation ratios for paired SEP and paired VEP recordings on the one hand and the estradiol or progesterone levels on the other hand (paired SEP: estradiol p = 0.544, r = 0.115; progesterone p = 0.959, r = 0.010; paired VEPs: estradiol p = 0.812, r = 0.045; progesterone p = 0.292, r = 0.199). In addition, we analysed for each participant a possible relationship between the changes of hormone levels during the menstrual cycle from low to high concentrations and the associated changes in paired-stimulation ratios. Likewise, this analysis failed to reveal any significant correlations (paired SEP: estradiol p = 0.803, r = − 0.070; progesterone p = 0.580, r = 0.155; paired VEPs: estradiol p = 0.752, r = 0.089; progesterone p = 0.565, r = 0.162). Conceivably, the lack of a relationship between paired-stimulation ratios and hormone levels is most likely due to the substantial interindividual variability of both hormone and excitability measures.

### Relation between paired-pulse suppression in somatosensory and visual cortex

The observation that higher estradiol levels affect excitability in both somatosensory and visual cortex imply a rather global action of estradiol on intracortical processing. We therefore analysed in how far paired-stimulation ratios recorded in both sensory modalities were coupled. Pearson’s correlation analysis revealed, however, that excitability in both areas was largely independent (p = 0.951, r = − 0.012). According to these data, participants characterized by low excitability in somatosensory cortex can show high excitability in visual cortex, and vice versa. However, independent of the individual hormone level, high levels of estradiol act to increase excitability in both the somatosensory and the visual cortex.

## Discussion

In the present study we investigated whether and to which extent minimal and maximal levels of estradiol and progesterone emerging during the menstrual cycle modulate the excitability of the primary somatosensory and visual cortex. As a marker of cortical excitability, we used paired-stimulation protocols in combination with recordings of SEPs and VEPs. Paired-pulse suppression (PPS) describes the suppressive effect of the first stimulus on the second one, which is quantified as the ratio of the amplitude of the second response divided by the first response amplitude. We found that during periods of low estradiol levels paired-pulse suppression was enhanced in both sensory modalities. In contrast, during high estradiol levels paired-pulse suppression was reduced indicative of high excitability. No comparable differences were found for low and high progesterone levels.

Our study was designed to assess paired-pulse suppression in a cohort of female participants at those time points within their menstrual cycle, where the levels of estradiol and progesterone were minimal and maximal. This procedure differs from previous reports about possible effects of gonadal hormones on cortical excitability^17,35–39^, which assessed paired-pulse suppression in a cohort of female participants at fixed time points within their menstrual cycle. By this, we were able to identify clear differences of paired-pulse suppression between episodes of low and high estradiol levels.

While our data show a clear dependence of paired-pulse suppression from estradiol levels, in contrast to reports from motor cortex^35,39^ our data failed to show any effects of high progesterone. These discrepancies might be due to modality-specific effects. However, the anatomical structure and functional organization of primary sensory and primary motor cortical areas, which subserve the input and output system, differ substantially. SEP and VEP recordings represent the activation of neurons of a sensory area following stimulation of the natural afferent pathway, while motor evoked potentials (MEPs) are generated by an artificial transsynaptic stimulation of pyramidal tract neurons. Accordingly, excitability parameters of paired-stimulation techniques in motor and sensory system are not easily transferable, which hinders a comparison of the physiological processes in an afferent sensory and an efferent motor system. However, there is the communality in that the underlying mechanisms mediating paired-pulse suppression in sensory and motor cortical areas are GABA mediated^26,42^.

In our study, we were interested in excitability fluctuations occurring in sensory cortices. While many studies by means of paired-stimulation paradigms have looked into intracortical inhibition in somatosensory and visual cortex, to our knowledge, this is the first study addressing possible hormonal influences. Our data demonstrate that excitability in somatosensory and visual cortex is jointly up- and down regulated during phases of low- and high estradiol levels. These observations, together with the available results on motor cortex excitability, provide further evidence that cortical excitability is globally modulated by gonadal hormones levels through the menstrual cycle.

Our data demonstrated that during elevated estradiol levels cortical excitability as marked by reduced paired-pulse suppression was increased. However, no differences in excitability were observed between phases of low and high progesterone levels, although the hormone levels differed by a factor of more than 10. Though not significant, the values for paired-pulse suppression in somatosensory cortex were even higher indicative for enhanced excitability. This observation is difficult to reconcile with the cellular findings, showing that estradiol and progesterone exhibit opposite effects on excitatory and inhibitory receptors^9,20^. There is agreement that estradiol augments N-methyl-D-aspartate- (NMDA)-mediated glutamate receptor activity and suppresses GABA inhibitory inputs^10,11^. In contrast, progesterone induces indirect effects after being metabolized to neuroactive steroids, augments GABA-mediated neurotransmission through its action at GABA_A_ receptors^43^ and counteracts neural excitation by suppressing excitatory glutamate responses. It therefore can be expected that progesterone modulates paired-pulse suppression.

In Fig. 7, the presented estradiol levels were measured at the time of minimal and maximal progesterone levels. While estradiol levels (123.5 ng/l) at the time of maximal progesterone (~ day 20) are lower than those measured at maximal estradiol (174.9 ng/l, ~ day 14), they are still significantly above the estradiol levels (32.7 ng/l) measured at the time point of minimal estradiol (~ day 4). We therefore suggest that high progesterone might exert an inhibiting effect on excitability in line with cellular studies and with data from Smith et al.^35^, but that the levels of estradiol that are very high at the same time counteract this effect, resulting in an overall lack of effects. Larger cohorts are needed to further explore these possibilities.

The mechanisms that underlie paired-pulse behaviour, which are often subsumed as short-term plasticity^44^, have been extensively studied theoretically and experimentally, but many aspects still remain elusive. For example, studies in rat auditory cortex have shown that at short ISIs forward suppression is largely controlled by GABA_A_ receptor-mediated inhibition^45^. However, other studies have shown that GABA_B_ receptors are likewise involved in the control of paired-pulse suppression^46^. In addition to the contribution of inhibitory transmitter systems, metabotropic glutamate receptors play a role in mechanisms mediating the paired-pulse phenomenon^47^. Another central aspect relates to vesicle depletion of calcium buffers and changes of release probabilities of excitatory synapses^44^.

Using drug applications of pharmacological modulators interfering with GABAergic neurotransmission such as the GABA_A_ agonist lorazepam, direct evidence for a GABAergic role controlling cortical excitability was provided for human motor cortex^42,48^ and somatosensory cortex^26,49^.

Because of differences in the paired-pulse behaviour between cortical and subcortical cells, it has been argued that inheritance of thalamic response properties is unlikely to account for long-lasting forward suppression^45^, on the other hand, thalamic neurons have been shown to display a complex form of adaptation following paired stimuli^50^. For human subjects, based on multichannel SEP-recordings after paired median nerve stimulation, it has been shown that paired-pulse suppression is generated at least rostral to the brainstem nuclei^24^. Taken together, the available data underlying paired-pulse mechanisms suggest a strong GABAergic involvement, which is most likely specific for processing stages in primary cortices and beyond.

Previous findings addressing effects of paired-pulse suppression in the context of plastic reorganisation associated with learning processes consistently showed that alterations of paired-pulse suppression were due to changes of second response amplitudes with no changes of the first amplitude. This observation has been interpreted that in contrast to the intracortical processing the afferent input remains largely unchanged. Our data about an impact of gonadal hormones over the menstrual cycle on single-stimulation EPs showed a comparable pattern. While the amplitude of the second response peak was affected, no comparable effects were found for single-stimulation EPs. These results indicate that estradiol and progesterone affect largely intracortical processing with little modulatory effects on afferent input strength.

As to the investigation of gonadal hormones on visual cortex processing, to our knowledge, there are no reports using pattern onset/offset VEPs. Previous studies investigating the impact of female hormones on VEPs had revealed discrepant results. In a study using flash-light evoked potentials, during the luteal phase longer latencies and higher amplitudes were reported^51^. In contrast, in another study that employed pattern reversal VEPs shorter latencies were found, which was interpreted as a signature of the facilitating effect of estradiol^52^. On the other hand, Resende et al.^53^ found reduced latency of the P100 peak during the luteal phase and stated that progesterone could improve neuronal excitability. Avitabile et al.^54^ found during the luteal phase a significant reduction in P100 latency compared with the follicular phase. They assumed that using different visual stimuli lead to contradicting results and ambiguous interpretations.

In our study, we have used pattern onset/offset VEPs to allow a reliable assessment of paired-stimulation suppression^40^. The approach of pattern onset/offset VEPs is less sensitive to confounding factors such as poor fixation, eye movements or deliberate defocus than pattern reversal VEP. In addition, the pattern onset/offset evoked VEPs differ clearly from VEPs evoked by other stimulation types, which has led to a different nomenclature termed C1, C2 and C3 (cf.^55^). Taken together, there are no consistent data on the impact of gonadal hormones on VEPs. Moreover, a comparison of different studies using different forms of stimulation is difficult.

In the group of female participants, the amount of paired-pulse suppression was significantly different for the time points of minimal compared to maximal estradiol levels (Fig. 4). When comparing these two time points with the average values found in the group of age-matched men, during maximal estradiol no differences between the groups of men and female were detectable. Only during low estradiol levels, the paired pulse suppression values for paired SEPs were significantly lower indicating less excitability compared to men. Because the same analysis failed to reach significance levels for VEPs, this observation must be interpreted with caution. Further studies are need to explore in how far excitability levels in men and in women during high estradiol levels are comparable. However, a similar observation has been made by Inghelleri et al.^37^, who suggested that in men testosterone and its metabolites might constantly exert a modulatory action on cortical excitability comparable to the transient effects of estradiol^37^.

The main results of this study show that low and high estradiol levels as observed during normal fluctuation across the menstrual cycle, modulate cortical excitability in a similar way in both the somatosensory and the visual cortex. It was found that high estradiol levels were associated with high intracortical excitability and vice versa. In contrast to estradiol, no comparable modulating effects could be found for progesterone levels. It is possible that the high estradiol levels present at high progesterone might have confounded a suppressive effect that could have been expected due to the inhibitory action of progesterone known at a cellular level. Another unexpected finding was that cortical excitability was comparable in men and female participants during high estradiol levels suggesting that other hormones such as testosterone might be involved in regulation of cortical excitability. Taken together, our data demonstrate that fluctuating hormone levels can severely impact cortical processing and cortical plasticity processes via their ability to interfere with the balance between excitation and inhibition.

In the current study we find that estradiol levels affect intracortical inhibition in somatosensory and visual cortex, but we did not find comparable effects for progesterone. As some previous studies performed in motor cortex^35,39^ had provided evidence for a progesterone action, these differences might be attributable to modality- or area-specific differences. However, under real-life conditions, the action of an individual compound can never be studied in isolation. Instead, there will always be a complex orchestrated mix of influences from the entire spectrum of hormones and neuroactive substances. The differentiation between estradiol and progesterone effects therefore needs to be investigated in the future. The possibility that high estradiol levels counteract the assumed suppressing effects of progesterone on excitability could be tested using specific anticonceptiva such as progesterone-only pills.

## Methods

### Subjects

Fifteen young adult normal-cycling women (27.1 ± 2.5 years; mean ± standard deviation) and fifteen men as a control-group (25.7 ± 2.8 years; t-test p = 0.183) participated in this study. All participants were right-handed as determined by the Edinburgh-Inventory^56^. All participants were without any disease or affection of the central or peripheral nervous system. Exclusion criterions were intake of any medication affecting the central nervous system within the last 6 months, and the use of any hormone-based contraception within the last 6 months. In addition, females who were pregnant or breastfeeding were not considered. Participants were recruited by announcements via the bulletin board at the “Ruhr-University of Bochum”, and by flyers distributed at the University Hospitals of Bochum. The study was approved by the Ethics Committee of the Medical Faculty of Ruhr-University of Bochum (Ref 4409–12) and was performed in accordance to the Declaration of Helsinki. All participants gave written informed consent before inclusion into the study and were paid for their participation.

### Paired median nerve stimulation evoked somatosensory potentials (SEPs)

Somatosensory evoked potentials were recorded following single and paired electrical stimulation of the right median nerve. The procedure was similar to previous studies using paired-stimulation in somatosensory system^24,27,28^. For median nerve stimulation we used a block electrode placed on the wrist of the right hand. Single (SS) and paired-stimuli (PS) were applied alternating with a repetition rate of 2 Hz using a Digitimer DS7A Current Stimulator (Digitimer Ltd, Hertfordshire, UK) timed by a custom-built timing device.

(microcontroller board, Arduino). Stimulus duration was 0.2 ms and interstimulus interval (ISI) of the paired-stimuli was 30 ms. In a series of previous studies, we had used ISIs of 30 ms to demonstrate robust paired-pulse suppression^27,34,57^. According to reported recovery functions recorded in somatosensory cortex, pronounced paired-pulse suppression is present at ISI < 50 ms^27,34,58^. Sensory threshold was defined as the lowest intensity at which a prickling sensation was perceived in the median nerve supply area of the right hand. Stimulation intensity used for SEP recordings was adjusted individually in each subject to the twofold of the individual sensory threshold. In addition to that, the median nerve stimulation had to induce a small muscular twitch in the thenar muscle, otherwise the intensity was further increased until twitching was induced. During the recording session, participants seated in a comfortable chair and were instructed to relax but stay awake with closed eyes.

SEPs were recorded using an electrode placed over the left somatosensory cortex, 2 cm posterior to C3 (CP3), according to the international 10–20 system^59^. A reference electrode was placed over midfront (FZ) position. Data were recorded using a 32-channel-amplifier (Brain Amp, Brain Products, Germany, sampling rate of 5 kHz, bandpass filter between 2 and 1000 Hz), and were digitized in a PC running the BrainVision Recorder software package (Brain Products GmbH, Germany). Resistances were kept below 5 kW. We recorded a total of 800 stimulus-related responses, for each single- and paired- stimulation. Offline, SEP raw data were segmented in epochs from 30 ms before to 80 ms after stimulus onset for each single- and paired- stimulation and baseline corrected, movement and muscle artifacts (amplitudes ≥ 100 μV) were rejected, and averaging was performed. Peak-to-peak amplitudes of the cortical N20 and the P25 response components for the first and second paired-stimulation were analysed. To eliminate confounds from superposition, we analysed paired-pulse suppression after linear subtraction of the cortical response after single stimulation (second amplitude after subtraction = A2s) and referred it to the first response of the paired-stimulation before linear subtraction (A1) (Fig. 1A). Paired-pulse suppression was expressed as an amplitude ratio (A2s/A1) of the subtracted second (A2s) and first (A1) amplitude.

### Paired-checkerboard stimulation evoked visual potentials (VEPs)

Subjects were seated in a darkened room. At an observation distance of 50 cm stimuli were displayed on a cathode ray tube spanning 23° × 17° of visual angle (CRT frame rate 75 Hz, pixel resolution of 800 × 600). Subjects were instructed to relax and to focus binocularly on a small dim fixation mark in the centre of the display. The stimulation setting was the same as described previously^60,61^. The experimental paired-stimulation paradigm consisted of checkerboard patterns with 36% contrast and with a mean luminance of 16 cd/m^2^ and a check size of 0.5°. The stimuli to evoke VEPs were generated by the EP2000-System^62^. Paired stimuli were presented at a stimulus onset asynchrony (SOA) of 93 ms. Previous studies had shown that a SOA of 93 ms revealed the most efficient PPS^40,60^. In this configuration and corresponding to the frame rate of the tube, the first stimulus appeared for one frame (13.33 ms), followed by presentations of frames containing a homogenous grey background without a change in the mean luminance. The second stimulus appeared after 6 times of the frame interval of 13.33 ms for the SOA of 93 ms to avoid temporal aliasing (see also^63^). Both the single- and the paired-stimulation condition were presented in four successive cycles of 10 stimuli, resulting for a total of 40 sweeps per condition.

For recording VEPs, Ag/AgCl electrodes were placed at Oz and referred to FPz, according to the international 10–20 system^59^. VEPs were recorded using a 32-channel-amplifier (BrainAmp, BrainProducts, Germany, sampling rate 5 kHz, band-pass filtering between 2 and 1000 Hz, resistances below 5 kW) and stored for offline analyses. Evoked potentials after single- and paired-stimulation were analysed in epochs from 200 ms before and 400 ms after the stimulus onset, baseline corrected to the pre-stimulus and averaged. Signals exceeding 140 µV were rejected as artifacts and not counted for the stimulation sequence. The responses of the first and second stimulus are characterized with A1 and A2. The positive and negative components of the responses are denoted with the term C (Fig. 1B). To characterize the paired-stimulation response, the amplitude difference of the C1_1_ (positive peak about 100 ms after stimulus onset^55^ and the C2_1_ (negative peak following C1) was measured. To eliminate confounds from superposition, we subtracted the response of the single-stimulation from the response of the paired-stimulation (A2s). Paired-stimulation was expressed as a ratio (A2s/A1) of the amplitudes of the second (A2s) and first (A1) stimulus.

### Experimental setting

The study was designed as a randomized and controlled trial. Each gender group consisted of 15 subjects, balanced to age. At each time of measurement, recordings of both single- and paired-stimulation SEPs and VEPs were performed. To obtain the minimal and maximal levels of estradiol and progesterone, women were tested at day 2–4 (T1, follicular phase), day 10–11 (T2, follicular phase), day 14 (T3, ovulation), and day 20–22 (T4, luteal phase) of their menstrual cycle. To avoid sequence effects, women were enrolled at different times of menstrual cycle. Blood samples were taken in women to analyse estradiol and progesterone levels at each time of measurement. The male control-group was tested in weekly interval during a period of four weeks.

### Statistics

The data were analysed using repeated measurement analysis of variance (rmANOVA) with within-subject factor “time” and between-subject factor “gender” to assess differences between hormone levels, between different points of measurements characterizing the excitability parameters, and to compare paired pulse data in men and women during different hormone levels. Significance was assumed at the P = 0.05 level. When appropriate, post-hoc two-sided t -tests were additionally conducted. The significance level was adjusted by dividing by the number of comparisons (Bonferroni correction). All calculations were performed using IBM SPSS Statistics 26.0 software package. Pearson’s correlation coefficient was assessed to detect possible relationships between estradiol and progesterone levels during menstruation cycle in women on SEP- and VEP- paired-stimulation ratio (A2s/A1) compared to control group. Significance was assumed at the P = 0.05 level.

## Data Availability

The datasets generated and analysed during the current study are available from the corresponding author on reasonable request.
